# Nocturnal blood pressure dipping and left ventricular hypertrophy among hypertensive outpatients in a Ghanaian hospital

**DOI:** 10.4314/gmj.v58i3.7

**Published:** 2024-09

**Authors:** Frederick A Akoto, Abdul-Subulr Yakubu, Francis Agyekum, Alfred Doku, Joseph A Akamah

**Affiliations:** 1 Department of Internal Medicine, University of Ghana Medical Centre, Accra, Ghana; 2 Department of Internal Medicine, Tamale Teaching Hospital, Tamale, Ghana; 3 Department of Medicine and Therapeutics, University of Ghana Medical School, Accra, Ghana; 4 Department of Internal Medicine, Meharry Medical College, Nashville, USA

**Keywords:** Blood pressure dipping, Ghana, Hypertension, Left ventricular hypertrophy, Ambulatory blood pressure

## Abstract

**Objectives:**

To investigate the association between the extent of nocturnal systolic blood pressure decline and left ventricular hypertrophy in patients with primary hypertension who were receiving antihypertensive drug therapy.

**Design:**

This was a cross-sectional hospital-based study from November 2020 to March 2021.

**Setting:**

The study was conducted at the Polyclinic of Korle Bu Teaching Hospital, Ghana.

**Participants:**

Outpatients ≥18 years old with primary hypertension who were receiving antihypertensive drug therapy.

**Interventions:**

Each participant underwent a 24-hour ambulatory blood pressure monitoring and a transthoracic echocardiogram.

**Main outcome measures:**

Left ventricular hypertrophy and the extent of mean systolic blood pressure decline during sleep.

**Results:**

180 participants were recruited, comprising 110 (61.1%) females. The participants' mean (±SD) age was 57.6 ± 11.0 years. 80% had a non-dipping blood pressure pattern, and 43.9% had left ventricular hypertrophy. Uncontrolled office blood pressure was an independent predictor of left ventricular hypertrophy in these patients (AOR 2.010, 95% CI 1.048-3.855, p=0.036); however, a non-dipping nocturnal systolic blood pressure status was not (AOR 1.849, 95% CI 0.850-4.022, p=0.121). 61.1% had abnormal left ventricular geometry, with concentric hypertrophy being the predominant geometric pattern.

**Conclusion:**

Left ventricular hypertrophy and non-dipping nocturnal blood pressure were common in these hypertensive Ghanaian patients on antihypertensive therapy. Left ventricular hypertrophy was associated with uncontrolled office blood pressure but not the extent of nocturnal systolic blood pressure declines during a single 24-hour ambulatory blood pressure recording.

**Funding:**

None declared

## Introduction

Hypertension is a major public health issue in Ghana, being a leading cause of admissions and deaths in rural and urban populations.^1^–^3^ About a quarter of Ghanaians have hypertension. However, awareness and control rates remain poor.^1^,^2^ Left ventricular hypertrophy (LVH) is a common finding among hypertensives in indigenous black populations in sub-Saharan Africa.^4^–^6^ Among Ghanaian hypertensives, LVH is a common cardiac complication, with uncontrolled BP being one of its main predictors.^7^ Left ventricular hypertrophy occurs in response to several hemodynamic and non-haemodynamic variables and is an independent predictor of cardiovascular events in hypertensive patients.^8^

Blood pressure (BP) exhibits a physiological circadian variation during ambulatory blood pressure monitoring (ABPM).^9^ Blood pressure measurements obtained by ABPM are closely related to cardiovascular disease and have prognostic significance.^10^ Non-dipping nighttime BP in hypertensives, which indicates a longer exposure to a higher BP load during the 24 hours, has been associated with the development of LVH.^11^,^12^ Nighttime BP values are less affected by the variable interference of individual daytime activities. They are more reproducible than daytime BP and may accurately reflect the individual's usual or ‘basal’ BP status.^13^

Studies on the association between LVH and an impaired decline in blood pressure during sleep among hypertensives have produced mixed results.^14^ It has been suggested that nocturnal hypertension, rather than dipping status, was independently associated with left ventricular hypertrophy. The simultaneous presence of non-dipping status and nocturnal hypertension predicted a higher left ventricular mass and an enhanced cardiovascular risk.^15^ It is, however, clear that reliance on office blood pressure measurements alone may be an ineffective way of assessing BP control.^16^ Among apparently controlled hypertensive subjects, based on office blood pressure measurements alone, cardiac structural abnormalities persisted despite antihypertensive therapy.^17^ Hence, effective 24-hour BP control BP is key in preventing subclinical adverse cardiac remodelling.^12^

This study sought to assess the association between a non-dipping systolic BP pattern and the presence of LVH in an indigenous black population with primary hypertension who were on antihypertensive therapy. The presence of such an association may justify incorporating dipping status into a comprehensive assessment of BP control and provide an additional therapeutic target for hypertensive patients beyond achieving the BP goal.

## Methods

This was a cross-sectional study on outpatients with primary hypertension receiving drug therapy at the Korle Bu Teaching Hospital polyclinic. The study site had 7 consulting rooms in the outpatient department. Based on statistics for the year 2019, an average of 7 hypertensive patients were seen in each consulting room daily. Patients were selected as follows. One of the consulting rooms was selected by balloting on each alternate day (i.e. Mondays, Wednesdays, and Fridays). The patient folders were identified and assigned numbers. The numbers were placed in an opaque envelope, and three were drawn without replacement. Thus, 9 participants were recruited per week. Data was collected over 20 weeks, from November 2020 to March 2021.

Inclusion criteria were age ≥18 years, a diagnosis of primary hypertension, receipt of antihypertensive drug therapy, and whether BP was controlled or not. Patients with congestive heart failure, valvular heart disease, ischaemic heart disease, congenital heart disease, haemoglobinopathies, diabetes, stroke, established chronic kidney disease and night shift workers were excluded. A minimum sample size of 170 was required based on an estimated prevalence of LVH of 12.6% among non-dipping hypertensives taking antihypertensive medication, a 5% margin of error and a 95% confidence level.^18^

Demographic and clinical information were collected through verbal interviews and a review of their electronic medical records. Relevant laboratory results were reviewed to identify comorbidities, cardiovascular risk factors and any exclusion criteria. Participants were also examined for evidence of cardiovascular disease, and anthropometric measurements were taken. The BP was initially measured sequentially in both arms of the participants. Three consecutive BP readings were taken in the seated position at two-minute intervals in the arm with higher BP using an oscillometric sphygmomanometer after the participant had rested for 5 minutes.^19^ The BP was the average of the second and third readings. Office BP was considered uncontrolled if the average systolic BP was ≥ 140 mmHg or the average diastolic BP was ≥ 90 mmHg.^16^

A 24-hour ABPM was performed for each participant (Contec ABPM 50, Contec Medical Systems Co Ltd.) with the device set to obtain BP readings at 30-minute intervals during the day and hourly intervals at night. Patients were instructed to go about their normal routine, remain still during measurements, and keep a diary of their sleep time and time of waking up. Sleep diaries were used to define individualised awake and asleep periods. The validity of the measurements was based on recommendations from the European Society of Cardiology/European Society of Hypertension.^19^ The BP recordings were analysed to obtain mean 24-hour, mean awake and asleep systolic and diastolic BP. A dipping pattern was defined by a 10% or more decline in average systolic BP during sleep relative to daytime systolic BP. Those who demonstrated a 10-20% decline in average systolic BP during sleep relative to daytime systolic BP were considered “normal dippers” whilst a ≥ 20% decline in systolic BP during sleep identified “extreme dippers”. A non-dipping status was defined as a <10% decline in systolic BP during sleep.^20^ The threshold for uncontrolled 24-hour ABPM was an average systolic BP ≥ 130 mmHg or an average diastolic BP ≥ 80 mmHg. Patients with controlled 24-hour ABPM readings but uncontrolled office BP readings were considered to exhibit a “white-coat effect”, while those with an uncontrolled 24-hour ABPM reading but controlled office BP were classified as exhibiting a “masked uncontrolled hypertension”.^16^

A transthoracic 2 - 2-dimensional (2D) and 2D- oriented M-mode echocardiography was performed in the lateral decubitus position using a commercially available ultrasound machine (Vivid T9 ultrasound imaging system, General Electric Healthcare, USA) equipped with a 3.5 MHz phased-array transducer. The end-diastolic (LVIDd) and end-systolic (LVIDs) left ventricular internal diameters, the interventricular septal thickness (IVSd) and the posterior wall thickness (PWTd) in diastole were measured. Left ventricular mass was calculated using the Devereux modified American Society of Echocardiography cube method:^21^ Left ventricular mass (g) = 0.8 (1.04 [IVSd + LVIDd + PWTd)^3^ – (LVIDd)^3^]) + 0.6g. Left ventricular hypertrophy was present when left ventricular mass (g) indexed to height raised to the power 2.7 (h^2.7^)(LVMI) exceeded 51 g/h^2.7^ in both men and women.^22^ The Left ventricular geometry pattern was classified based on the LVMI and the relative wall thickness.^23^

### Data analysis

Continuous variables were expressed as their means and standard deviations, and categorical variables as frequencies and percentages. The Chi-square test was used to compare categorical variables between groups, whereas the independent sample Student's t-test was used to compare the means of two continuous variables. Univariate and multivariable logistic regression analysis was performed to identify the predictors of LVH. Variables tested included demographic characteristics, blood pressure, nocturnal BP dipping status, and other cardiovascular risk factors such as physical activity, smoking, and body mass index. Nocturnal BP dipping status and other variables that showed a significant association with LVH in the univariate analysis were included in the multivariate analysis. A *p*-value of <0.05 was considered statistically significant. Statistical analyses were performed using the Statistical Package for the Social Sciences version 21.0 software (SPSS, IBM Corporation, Armonk, NY, USA).

### Ethical considerations

The Institutional Review Board of the Korle Bu Teaching Hospital (KBTH-IRB /00066/2020) granted this study's ethical approval. All participants provided written informed consent.

## Results

All recruited participants were included in the analysis. A total of 180 participants were recruited, comprising 110 (61.1%) females. The mean age (±SD) was 57.6 ± 11.0 years, ranging from 25 to 81 years. The demographic and clinical characteristics of the study participants are summarised in Table 1. Calcium channel blockers, beta-blockers, angiotensin-converting enzyme inhibitors, angiotensin receptor blockers, and diuretics were the main classes of antihypertensive medication used. The average number of antihypertensive medications each participant took was 1.8 ± 0.7.

**Table I:** Baseline characteristics of study participants

VARIABLE	TOTAL (N=180)	MALE (n=70)	FEMALE (n=110)	*p-value*
**Age (years)**	57.6 ± 11.0	58.1 ± 10.9	57.2 ± 11.1	0.600
**BMI (kg/m^2^)**	28.8 ± 6.5	27.7 ± 6.6	29.9 ± 6.3	0.029*
**Office BP (mmHg)**				
**Systolic**	150.2 ± 22.1	153.1 ± 22.6	147.3 ± 21.5	0.080
**Diastolic**	92.6 ± 13.8	94.2 ± 14.9	91.0 ± 12.8	0.120
**Heart rate (beats/min)**	76.3 ± 13.1	75.5 ± 13.9	77.2 ± 12.4	0.420
**Alcohol use**	38 (21.1%)	21(30.0%)	17(15.5%)	0.020*
**Smoking status**				0.038*
**Current smoker**	2 (1.1%)	2 (2.9%)	0 (0.0%)	
**Non-smoker**	171 (95%)	63 (90%)	108 (98.2%)	
**Former smoker**	7 (3.9%)	5 (7.1%)	2 (1.8%)	
**Physical activity**				0.210
**Inactive (<30mim/week)**	154(85.6%)	56 (80.0%)	98 (89.1%)	
**Moderate (30-60min/week)**	23 (12.8%)	12 (17.1%)	11 (10.0%)	
**Active (60-150 min)**	3 (1.7%)	2 (2.9%)	1 (0.9%)	
**Duration of hypertension**				0.480
**<1 year**	16 (8.9%)	4 (5.7%)	12 (10.9%)	
**1-5 years**	71 (39.4%)	29 (41.4%)	42 (38.2%)	
**>5years**	93 (51.7%)	37 (52.9%)	56 (50.9%)	
**Number of antihypertensives**				0.940
**1**	67 (37.2%)	25 (35.7%)	42 (38.2%)	
**2**	83 (46.1%)	33 (47.1%)	50 (45.5%)	
**≥3**	30 (16.7%)	12 (17.1%)	18 (16.4%)	

BMI, Body mass index; BP, Blood Pressure. Values are presented as mean ± standard deviation or n%.

* p is statistically significant

One hundred and twenty (66.7%) of the participants had uncontrolled office BP measurements (≥140/90 mmHg). The 24-hour ABPM showed that 107 (59.4%) of the participants had uncontrolled BP, with 35 (19.4%) showing a white coat effect and 22 (12.2%) having masked uncontrolled hypertension.

One hundred and forty-four (80.0%) participants had a <10% decline in SBP during sleep (non-dippers). Thirty-six demonstrated an asleep SBP fall > 10% (dippers); 30 (16.7%) of these had a 10-20% SBP decline (normal dipping), whilst six (3.3%) had a ≥ 20% decline in SBP during sleep (extreme dipping). There were no significant differences in gender, age, BMI, office BP and timing of antihypertensive drug intake between dippers and non-dippers (Table 2).

**Table 2:** Comparison of patient characteristics based on dipping status

VARIABLE	TOTAL, N=180	Dippers, n=36	Non-dippers, n=144	*p-value*
**Female**	110 (61.1%)	20 (55.6%)	90 (62.5%)	0.445
**Males**	70 (38.9%)	16 (44.4%)	54 (37.5%)	
**Age (years)**	56.7 ± 22.3	55.2 ± 11.4	58.1 ± 10.8	0.155
**BMI**	28.74 ± 6.1	28.20 ± 5.5	29.28 ± 6.7	0.378
**Office systolic (mmHg)**	150.4 ± 19.3	151.7 ± 24.0	149.0±14.6	0.505
**Office diastolic (mmHg)**	93.19 ± 13.9	94.8 ± 14.5	91.6 ± 13.4	0.205
**Timing of antihypertensive intake**				0.370
**Morning**	161 (89.4%)	33 (91.7%)	128(88.9%)	0.628
**Evening**	61 (33.9%)	12 (33.3%)	49 (34.0%)	0.937

BMI, Body Mass Index; BP, Blood Pressure Values presented as mean ± SD or n (%)

Using an LVMI exceeding 51 g/h^2.7^ as the cutoff, 79 (43.9%) participants had LVH. Blood pressure and BMI were significantly higher among LVH patients (Table 3). Age, gender, duration of hypertension, nocturnal hypertension or dipping status were not significantly associated with the presence of LVH. When the analysis was repeated for the subgroup of patients with controlled office and 24-hour ABPM measurements, BP dipping status was not significantly associated with LVH (*p*=0.718 and *p*=0.506, respectively). One hundred ten participants (61.1%) had abnormal LV geometry (concentric remodelling, concentric hypertrophy, and eccentric hypertrophy), with concentric hypertrophy being the predominant abnormal LV geometric pattern (27.8%). Higher BMI (*p*=0.014) and BP (*p*<0.05) were associated with the presence of abnormal LV geometry.

**Table 3:** Comparison of clinical characteristics according to LV mass

VARIABLE	LVH, n=79 (43.9%)	No LVH, n=101 (56.1%)	*p-value*
**Age**	58.5 ± 10.6	56.8 ± 11.3	0.306
**Females**	44 (55.7%)	66 (65.3%)	0.188
**Males**	35 (44.35%)	35 (34.7%)	
**BMI**	30.9 ± 7.4	27.7 ± 5.4	0.001*
**OFFICE BP**			
**Systolic**	154.5 ± 25.0	145.7 ± 18.7	0.007*
**Diastolic**	93.8 ± 15.2	91.0 ± 12.3	0.155
**AVERAGE 24 HR BP**			
**Systolic**	134.3 ± 17.0	128.9 ± 12.5	0.016*
**Diastolic**	82.4 ± 11.7	79.8 ± 10.9	0.101
**AWAKE (Day) BP**			
**Systolic**	135.7 ± 17.5	130.5 ± 14.0	0.032*
**Diastolic**	82.4 ± 11.8	79.8 ± 10.7	0.138
**ASLEEP (Night) BP**			
**Systolic**	131.6 ± 18.4	126.4 ± 12.9	0.017*
**Diastolic**	78.0 ± 12.6	76.4 ± 10.4	0.295
**Dipping status (Dipper)**	12 (15.1%)	24 (23.8%)	0.154
**Duration of hypertension (>5 years)**	46 (58.25)	47 (46.5%)	0.078
**Nocturnal hypertension**	60 (75.9%)	64 (63.4%)	0.070

BMI, Body Mass Index; BP, Blood Pressure Values presented as mean ± SD or n (%)

* Statistically significant

Univariate and multivariable logistic regression analyses were conducted to identify the predictors of LVH and assess the association between BP dipping status and LVH (Table 4). Uncontrolled Office BP was an independent predictor of LVH in these patients (AOR 2.010, 95% CI 1.048-3.855, p=0.036). A non-dipping nocturnal systolic BP status was not associated with an increased risk of LVH (AOR 1.849, 95% CI 0.850-4.022, p=0.121).

**Table 4:** Independent predictors of LVH

	Univariate analysis		Multivariate analysis^a^	
	COR (95% CI)	*p-value*	AOR (95% CI)	*p-value*
**Uncontrolled Office BP**	1.934 (1.015-3.687)	0.045*	2.010 (1.048-3.855)	0.036*
**Non-Dipping BP**	1.740 (0.809-3.745)	*0.157*	1.849 (0.850-4.022)	*0.121*
**Nocturnal HTN**	1.826 (0.948-3.517)	*0.072*	-	-
**HTN>5 years**	1.602 (0.884-2.901)	*0.120*	-	-
**Alcohol use**	0.575 (0.267-1.237)	*0.157*	-	-
**Male gender**	1.500 (0.820-2.745)	*0.188*	-	-
**Age > 65 years**	1.016 (0.510-2.026)	*0.964*	-	-
**Obesity**	1.468 (0.794-2.714)	*0.221*	-	-
**Smoking**	0.499 (0.094-2.641)	*0.413*	-	-
**ACEI/ARB use**	1.189 (0.652-2.171)	*0.572*	-	-
**Physical inactivity**	1.299 (0.554-3.043)	*0.547*	-	-

AOR, adjusted odds ratio; ACE, angiotensin converting enzyme inhibitor; ARB, angiotensin receptor blocker; BP, systolic blood pressure CI, confidence interval; COR, crudes odds ratio; HTN, Hypertension; LVH, Left ventricular hypertrophy.

a Nagelkerke R^2^=0.048, p=0.036

* p-value is statistically significant

## Discussion

In this study among hypertensive patients on antihypertensive therapy, majority of the participants had uncontrolled office or ambulatory BP measurements with 80.0% demonstrating a non-dipping nocturnal SBP pattern. Left ventricular hypertrophy was present in 43.9%, with the predominant geometry being concentric hypertrophy. Uncontrolled office blood pressure was an independent predictor of LVH, while a non-dipping nocturnal systolic BP was not.

Several factors have been known to influence the development of LVH.^24^ In addition to the severity of hypertension and degree of BP control, race and genetics have been known to affect the prevalence of LVH in various populations.^25^ Variations and a lack of consensus on the appropriate method of normalising LV mass have also contributed to the reported differences in LVH in various studies. Indexing left ventricular mass to BSA has been shown to underestimate the prevalence of LVH compared to indexation to height raised to the power 2.7, especially among overweight and obese persons with hypertension.^26^,^27^

LVH was associated with uncontrolled office blood pressure (BP). Similarly, the mean 24-hour SBP, mean awake SBP, and mean asleep SBP tended to be higher in those with LVH. Ambulatory blood pressure correlates closely with cardiovascular events and is superior to office blood pressure for predicting individual cardiovascular risk in treated hypertensives.^28^ A non-dipping BP pattern is common in hypertensive patients and has been said to be a marker of high cardiovascular risk.^29^ There was no association between a non-dipping nocturnal systolic BP and the presence of LVH in these Ghanaian hypertensives on antihypertensive drug therapy. Since most of these patients had uncontrolled office and ambulatory blood pressure, the haemodynamic consequences of this high blood pressure might have blunted any effect that a non-dipping nocturnal BP alone may have on left ventricular mass. Advanced age, high salt intake, cardiovascular comorbidities, smoking, obesity, poor sleep quality and physical inactivity during the recording period have been shown to influence the circadian BP and nocturnal BP dipping status.^29^,^30^ However, the nocturnal decline of blood pressure has been shown to suffer from poor reproducibility. Among hypertensive patients on treatment, dipping and non-dipping phenotypes were found to be markedly unstable, with frequent shifts from one to another during four years of follow-up.^31^ Furthermore, studies have suggested that a non-dipping pattern in hypertensive patients may merely be a marker of high cardiovascular risk in these patients and may not by itself independently identify hypertensive patients with greater cardiovascular damage.^14^,^29^,^32^,^33^

More than half of the patients in this study had an abnormal LV geometry, with concentric LVH being the predominant pattern. In addition to cardiac mass, dynamic changes in LV geometry occur over time and have incremental prognostic significance.^34^ In this study, higher BMI and blood pressure were modifiable risk factors associated with the development of abnormal LV geometry.

This study had some limitations. The relatively small sample size and single-centre design limit the generalizability of the results. Also, the classification of BP dipping status was made based on a single 24-hour ABPM recording, which may be less reliable than one based on at least two concordant 24-hour ABPM recordings (>48 hours ABPM).^13^

## Conclusion

Among these hypertensive Ghanaians on antihypertensive therapy, the presence of LVH was associated with an uncontrolled office BP but not the extent of nocturnal systolic BP decline during a single 24-hour ABPM recording. This finding is reassuring to clinicians practising in an environment with limited access to ABPM, and office BP measurement remains the primary means of assessing BP control.
